# A de novo variant in the human *HIST1H4J* gene causes a syndrome analogous to the *HIST1H4C*-associated neurodevelopmental disorder

**DOI:** 10.1038/s41431-019-0552-9

**Published:** 2019-12-05

**Authors:** Federico Tessadori, Atteeq U. Rehman, Jacques C. Giltay, Fan Xia, Haley Streff, Karen Duran, Jeroen Bakkers, Seema R. Lalani, Gijs van Haaften

**Affiliations:** 1 Hubrecht Institute-KNAW and UMC Utrecht, 3584 CT Utrecht, The Netherlands; 2 Department of Genetics, Center for Molecular Medicine, University Medical Center Utrecht, 3584 CX Utrecht, The Netherlands; 3 Department of Molecular and Human Genetics, Baylor College of Medicine, Houston, TX USA; 4 Department of Medical Physiology, Division of Heart and Lungs, University Medical Center Utrecht, 3584 CX Utrecht, The Netherlands; 5 Present Address: New York Genome Center, New York, NY USA

**Keywords:** Disease genetics, Disease model

## Abstract

We report here a de novo missense variant in *HIST1H4J* resulting in a complex syndrome combining growth delay, microcephaly and intellectual disability. Trio whole exome sequencing (WES) revealed that the proband was heterozygous for a de novo c.274 A > G p.(K91E) variant in *HIST1H4J*, a gene not yet associated with human disease. The patient presented with profound intellectual disability, microcephaly, and dysmorphic facial features. Functional consequences of the identified de novo missense variant were evaluated in zebrafish embryos, where they affected general development, especially resulting in defective head organs and reduced body axis length. Our results show that the monoallelic p.K91E substitution on *HIST1H4J* underlies a human syndrome that is genetically and phenotypically akin to the *HIST1H4C*-associated neurodevelopmental disorder resulting from p.K91A and p.K91Q substitions in *HIST1H4C*. The highly overlapping patient phenotypes highlight functional similarities between HIST1H4J and HIST1H4C perturbations, establishing the singular importance of K91 across histone *H4* genes for vertebrate development.

## Introduction

The importance of chromatin regulation is reflected by the ever-growing literature on human diseases caused by genetic alterations in histone-modifying complexes or histone genes [1–4].

We recently reported monoallelic, dominant pathogenic variants affecting lysine 91 (p.K91A or p.K91Q) in HIST1H4C (RefSeq NM_003542.3) causing a severe neurodevelopmental syndrome. We pinpointed the cause of the disorder to perturbation of early developmental stages due to the accumulation of DNA damage, genomic instability, and cell cycle delay [4].

HIST1HJ (RefSeq NM_021968.3) and HIST1H4C are two of the fifteen human genes encoding histone H4. While there are differences in the coding sequences of *H4* genes, they all encode an identical H4 protein [5]. Here we present data establishing that a de novo, dominant variant resulting in the substitution of lysine 91 by glutamic acid in *HIST1H4J* mirrors the phenotype previously reported for *HIST1H4C-*related disorder. The remarkable phenotypical overlap with HIST1H4C K91 patients [4] and functional data obtained in zebrafish provide compelling evidence that the mutated HIST1H4J K91 is causative for the proband’s neurodevelopmental disorder.

## Materials and methods

### Patient genetic investigation

The study was performed following the ethical guidelines for research involving human subjects and was approved by the Institutional Review Board at Baylor College of Medicine. Written informed consents were obtained from the participating family members. The proband was seen at Texas Children’s Hospital by SRL and was referred for clinical trio WES. Trio WES was performed at Baylor Genetics Laboratories as previously described [6, 7].

### Fish lines and husbandry

Tübingen longfin zebrafish were kept in standard laboratory conditions [8]. Animal experiments were approved by the Animal Experimentation Committee of the Royal Netherlands Academy of Arts and Sciences.

### Expression assay in zebrafish embryos

Capped mRNA microinjections were carried out essentially as described in [4]. Human cDNA encoding for HIST1H4J (RefSeq NM_021968.3) was used as template for single site mutagenesis with primers Hist1H4J_K91E_F: 5′-gtctacgcgctcgagcgccagggcc-3′ and Hist1H4J_K91E_R: 5′-ggccctggcgctcgagcgcgtagac-3′.

### Imaging

Live phenotypical assessment of 28 hpf zebrafish embryos was carried out on a Zeiss StemiSV6 stereomicroscope (Carl Zeiss AG, Oberkochen, Germany). Imaging was performed using a Zeiss Axioplan brightfield microscope (Carl Zeiss AG) and a Leica DFC420C digital microscope camera (Leica Microsystems, Wetzlar, Germany).

## Results

### Patient report

**Fig. 1:**
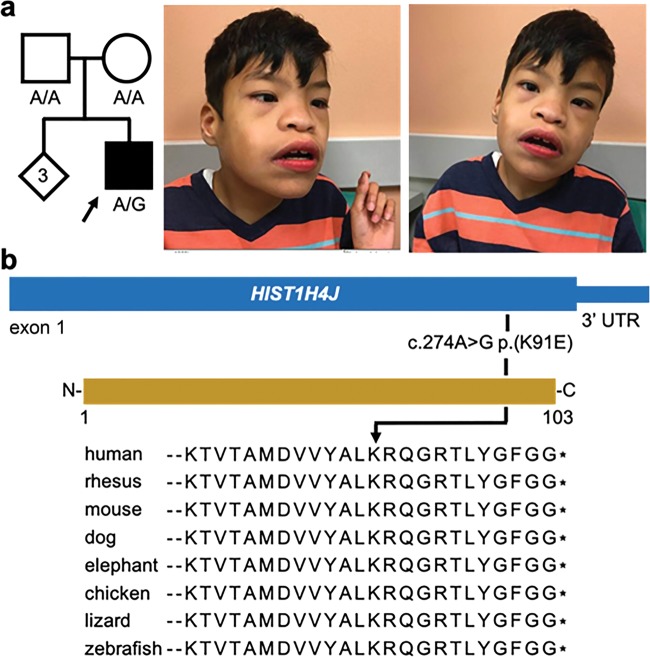
De novo missense variant identified in *HIST1H4J*. **a** Pedigree and photographs of the proband. The tilted square refers to unspecified sex. **b** Location of the de novo missense variant at gene and protein level and alignment of H4 residues demonstrating that K91 as well as surrounding residues are highly conserved across species. At the genomic level, this A > G substitution is located at chr6:27792176 (hg19). See main text for more information on the nomenclature

The patient was a 14-year old Hispanic male with profound intellectual disability. He was the product of a full term pregnancy with limited prenatal care. His birth weight was 2.3 kg. He was born with hypospadias, which was surgically repaired. Growth parameters remained <3rd percentile throughout his medical evaluation. He was globally delayed and hypotonic. He sat unassisted at 9 months of age and walked independently around 4 years of age. He had significant language delay and was diagnosed with pervasive developmental disorder. His additional diagnoses were oculomotor apraxia (OMA) and moderate angle left esotropia. At the age of 14 years, he was nonverbal. His height was 125.3 cm (−4.64 SD), weight was 20.7 kg (−4.13 SD), and head circumference was 49.4 cm (−3.32 SD). Dysmorphic features included upslanting palpebral fissures, hypertelorism, periorbital fullness, arched eyebrows, flat nasal bridge, wide mouth with downturned corners, and short philtrum (Fig. 1a). He had muscle wasting involving the upper and lower extremities, slender hands, and flat feet. Echocardiogram and renal ultrasound evaluations were normal. Brain MRI showed mild prominence of supratentorial sulci and cisterns. His laboratory work-up included very long chain fatty acids, CPK, lactate, and DNA analysis for Fragile X, which were all normal. Chromosomal microarray showed a paternally inherited 207 kb gain involving *KCNV1* on chromosome 8q23.2. *MECP2* sequencing was normal.

### Identification of the c.274 A > G p.(K91E) variant in HIST1H4J by WES

Analysis of trio WES data didn’t reveal any variant affecting or likely affecting known disease-associated genes that could explain the proband’s phenotype. Potentially disease-causing variants in maternally or paternally inherited copies of *KCNV1* were not identified.

However, the proband was found to be heterozygous (110 mutant vs 138 reference reads) for a de novo variant [chr6:27792176 A > G (hg19), c.274 A > G; p.(K91E)] in *HIST1H4J*, a gene hitherto not associated with a human disease. The c.274 A > G variant was neither present in his biological parents nor in control databases such as ExAC or gnomAD. Moreover, the affected K91 residue is extremely well conserved across species (Fig. 1), and the effect of the variant is predicted to be deleterious (SIFT) and possibly damaging (PolyPhen-2; http://genetics.bwh.harvard.edu/pph2/). No additional de novo variants were detected in the proband.

One homolog of *HIST1H4J*, *HIST1H4K*, is located just 6.7 kb away on the short arm of chromosome 6. While the coding sequences of 13 H4 genes display substantial variation with that of HIST1H4J (Fig. S1), *HIST1H4J* and *HIST1H4K* share an identical open reading frame and differ only in their 3′UTR sequence (Fig. S2). Visual analysis of the sequencing bam files confirmed that sequence reads containing the de novo A > G variant indeed originated and mapped back exclusively to the *HIST1H4J* locus (Fig. S2).

### Functional modeling of HIST1H4J K91E in zebrafish

**Fig. 2:**
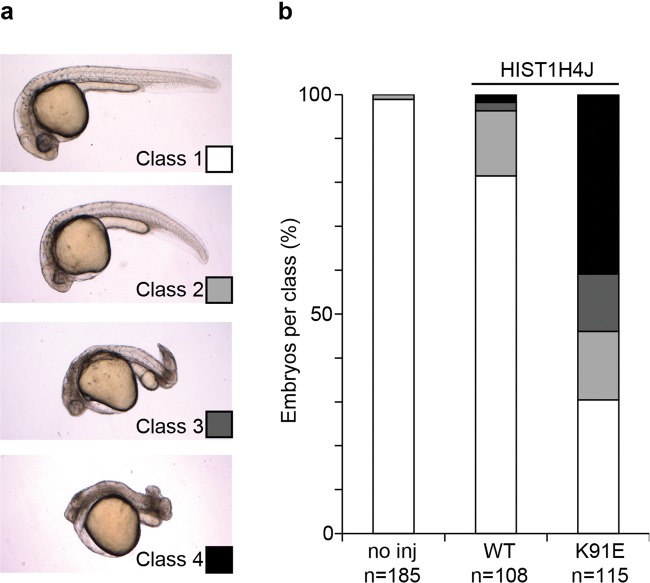
The K91E substitution on HIST1H4J induces early severe developmental defects in zebrafish embryos. **a** Phenotypes observed in zebrafish embryos at 28 h post fertilization. Wildtype HIST1H4J (WT) and K91E mRNA was microinjected at the 1-cell stage. Class 1 embryos display normal development, class 2 embryos display mild shortening of the body axis and delayed head development. Class 3 embryos have severely defective head development and a shortened AP body axis, with abnormal posterior development. In Class 4 embryos head structures and somites are largely absent. **b** Histogram presenting the percentage of observed embryos in each class for each category. no inj non-injected control. The data presented were collected over three independent biological and technical experimental replicates

We tested the HIST1H4J K91E variant for dominant effects on the development by microinjecting synthetic mRNA in zebrafish embryos (Fig. 2). A loss of function effect was not considered because of the presence of multiple loss of function variants in a range of histone H4 genes in the healthy population (Gnomad, [9, 10]).

Analysis at 28 hpf revealed that while the expression of WT HIST1H4J had only a very mild effect on the embryonic development, the expression of HIST1H4J K91E had a clear signature on the structural development of zebrafish embryos (Fig. 2), which is reminiscent of the previously reported phenotype for HIST1H4C K91 variants [4]. Defective development of head structures such as the brain and eyes, faulty body axis growth, and a dysmorphic tail were observed, which are all features evocative of the proband’s microcephaly and short stature.

## Discussion

We describe here a novel, dominant neurodevelopmental disorder associated with the substitution K91E on the *HIST1H4J* gene. The proband of this study presented with profound intellectual disability, microcephaly, and dysmorphic facial features. Trio WES analysis revealed that the proband was heterozygous for a de novo c.274 A > G p.(K91E) variant in *HIST1H4J*.

The proband’s clinical features, including his craniofacial dysmorphisms were strikingly similar to those reported previously in the patients with *HIST1H4C* variants (Table 1; [4]). While all patients shared general impaired neurodevelopment, growth parameters and distinctive craniofacial features, one of the features distinguishing the proband of this study was OMA, a condition characterized by defective, or absent voluntary, or attraction eye movements [11]. Since trio WES did not reveal any variants likely affecting the OMA-related genes, we concluded that this phenotype was likely related to the *HIST1H4J* change in the proband.

**Table 1:** Comparison of clinical and genetic findings in patients with variants of *HIST1H4C* and *HIST1H4J*

	*HIST1H4J*	*HIST1H4C* (pt. 1)	*HIST1H4C* (pt. 2)	*HIST1H4C* (pt. 3)
cDNA change	c.274 A > G	c.274 A > C	c.275 A > G	c.275 A > G
Effect	p.K91E	p.K91Q	p.K91R	p.K91R
Age at last visit	13 years	7 year	13 years	11 days
Gender	Male	Female	Female	Female
Ethnicity	Hispanic	Caucasian	Caucasian	Caucasian
Microcephaly	✓	✓	✓	✓
Hypotonia	✓	✓	✓	No
Developmental delay	✓	✓	✓	✓
Growth retardation	✓	✓	✓	NA
Intellectual disability	✓	✓	✓	NA
Brain MRI findings	Mild prominence of the supratentorial sulci and cisterns	Reduction in white matter bulk	Normal	NA
Ophthalmologic	Oculomotor apraxia, moderate angle left esotropia	Myopia, squint	Amblyopia of the left eye, small papillae, refraction anomaly, convergent strabismus of the left eye	
Craniofacial features	Upslanting palpebral fissures, hypertelorism, periorbital fullness, flat nasal bridge, wide mouth, short philtrum	Upslanting palpebral fissures, bifid flat nasal tip, median ridge on philtrum, wide mouth, hypertelorism, ptosis, exorbitism	Upslanting palpebral fissures, bifid flat nasal tip, median ridge on philtrum, wide mouth, hypertelorism, asymmetric eyes, periorbital fullness	Upslanting palpebral fissures, bifid flat nasal tip, retrognathia
Foot ray anomaly	Unknown	✓	✓	✓
Other features	Pervasive developmental disorder, hypospadias	Secundum atrial septal defect, small kidneys with lack of cortico-medullary differentiation and simple cysts, high pain threshold	Psychotic, lordosis, cutis marmorata, seizures	

The c.274 A > G variant detected in the proband results in the substitution of a lysine (K) by glutamic acid (E) at position 91 on the *HIST1H4J* gene. Lysine 91 posttranslational modifications include acetylation and monoubiquitination [12, 13] which play important roles respectively in chromatin assembly and stability [13, 14] and protection against DNA-damaging agents [12]. Since the acquired glutamic acid cannot be monoubiquitinated, and given its negative charge, the HIST1H4 K91E substitution presented here is likely to result in the genomic instability as described previously for substitutions at lysine 91 on *HIST1H4C* [4]. In addition, similarly to *HIST1H4C*, *HIST1H4J* is relatively well expressed in early human embryos and human embryonic stem cells [15], both systems with relatively short cell cycle time and consequently sensitive to perturbation of the cell division rate.

The discovery of the *HIST1H4J* syndrome described here shows that K91 variants are not just specific to HIST1H4C, but the substitution of K91 in other genes encoding the same H4 protein could also cause this recognizable neurodevelopmental syndrome. This was an important, outstanding question after the discovery of the *HIST1H4C*-associated neurodevelopmental disorder [4]. Clearly, variants in epigenetic pathways underlie both developmental syndromes and oncogenesis. As the abundance and nonrandom presence of histone variants is becoming increasingly evident in cancer [16] (and references therein), turning our attention to histone genes could provide us with the opportunity to resolve, at the genetic level, more yet unexplained developmental syndromes.

## Supplementary information

Supplementary material

## Data Availability

The genetic and phenotypical data were submitted to the Leiden Open Variation Database (LOVD; http://www.lovd.nl/3.0/home) as submission ID 00266138.
