# Supplementary material for: A de novo variant in the human HIST1H4J gene causes a syndrome analogous to the HIST1H4C-associated neurodevelopmental disorder
Source: Eur J Hum Genet. 2019 Dec 5;28(5):674–8. doi: 10.1038/s41431-019-0552-9 (PMC7171094; doi:10.1038/s41431-019-0552-9)
Supplement: Supplementary file 1 — Supplementary material [file 41431_2019_552_MOESM1_ESM.docx]

**Supplementary Information Tessadori, Rehman et al.**

**Note on Nomenclature:**

HGVS Variant nomenclature would refer to the residue studied here as HIST1H4J K9**2** [chr6:27792176 (hg19), c.274A>G p.(K9**2**E)] . However, it is identical to that described previously in [4], in which Molecular Biology nomenclature was utilized, excluding the first post-translationally removed methionine. Hence we have followed Molecular Biology nomenclature in this study as well.

**Figure S1**

**
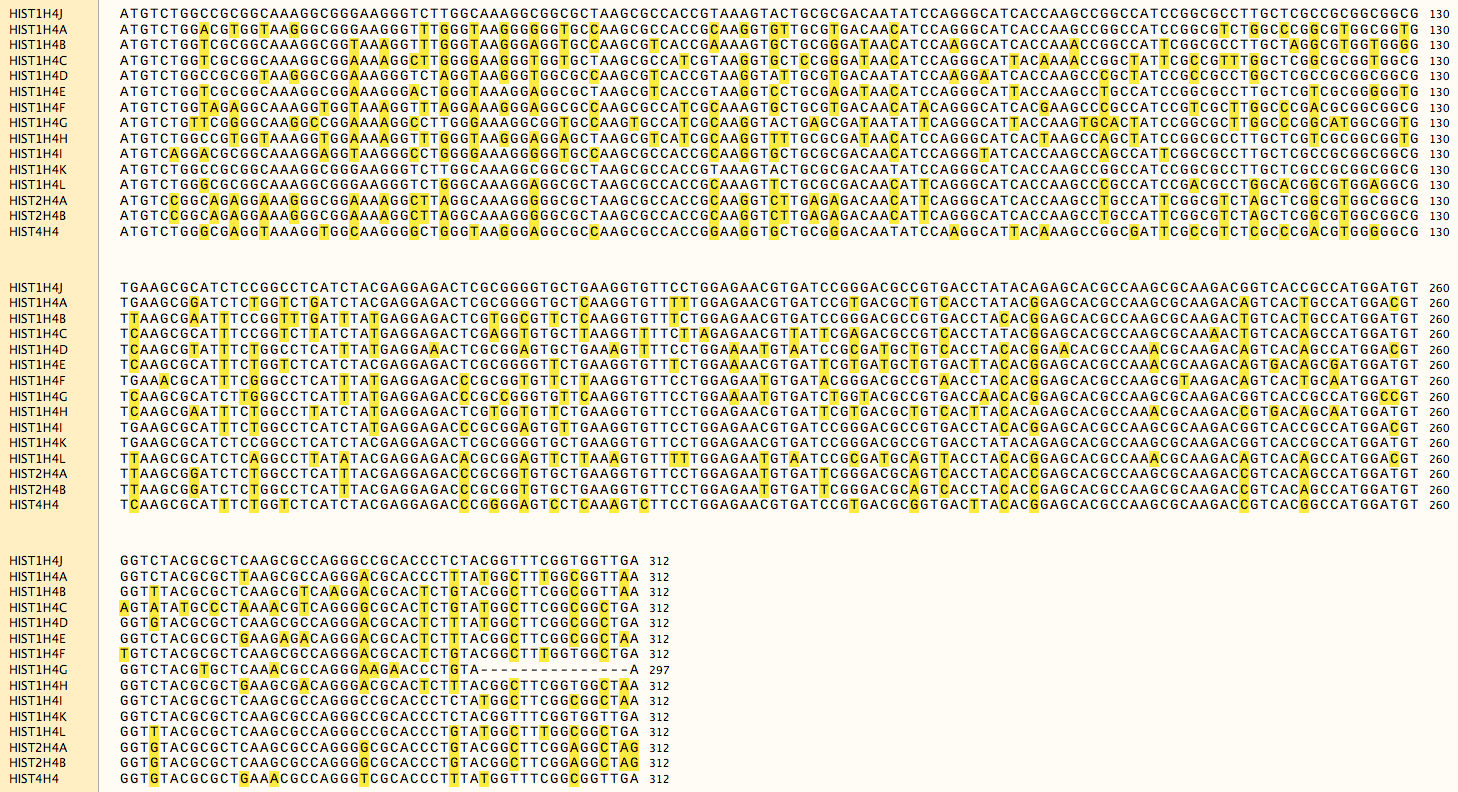
**

**Figure S1. Coding sequence alignment for human H4-encoding genes.** Coding sequence for HIST1H4J is taken as reference sequence. Nucleotides in other H4 coding sequences differing from the reference sequence are highlighted in yellow.

**Figure S2**

**a**


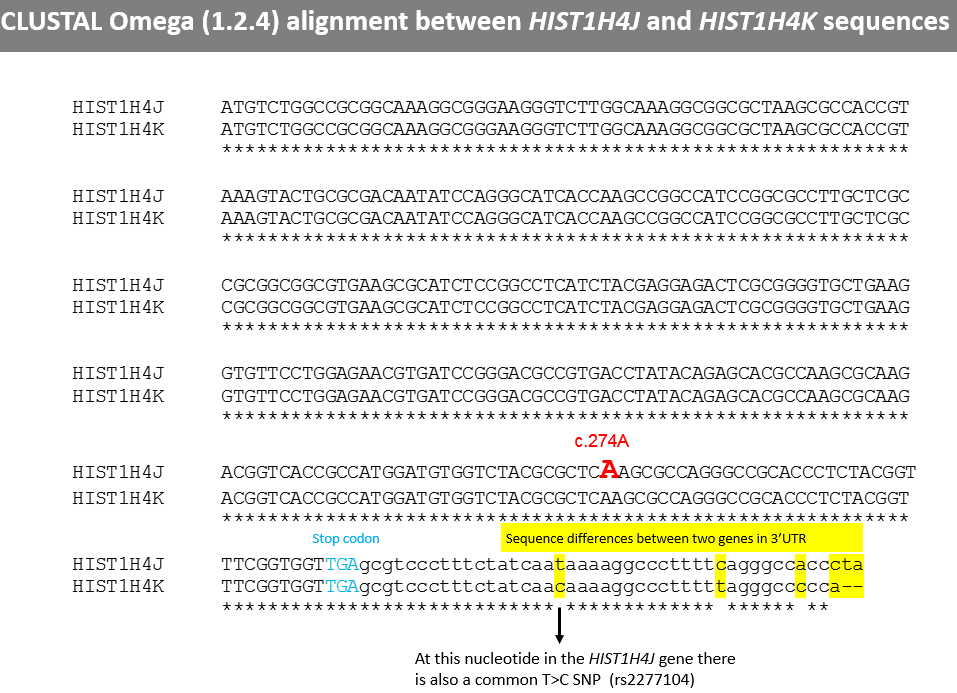


**b**


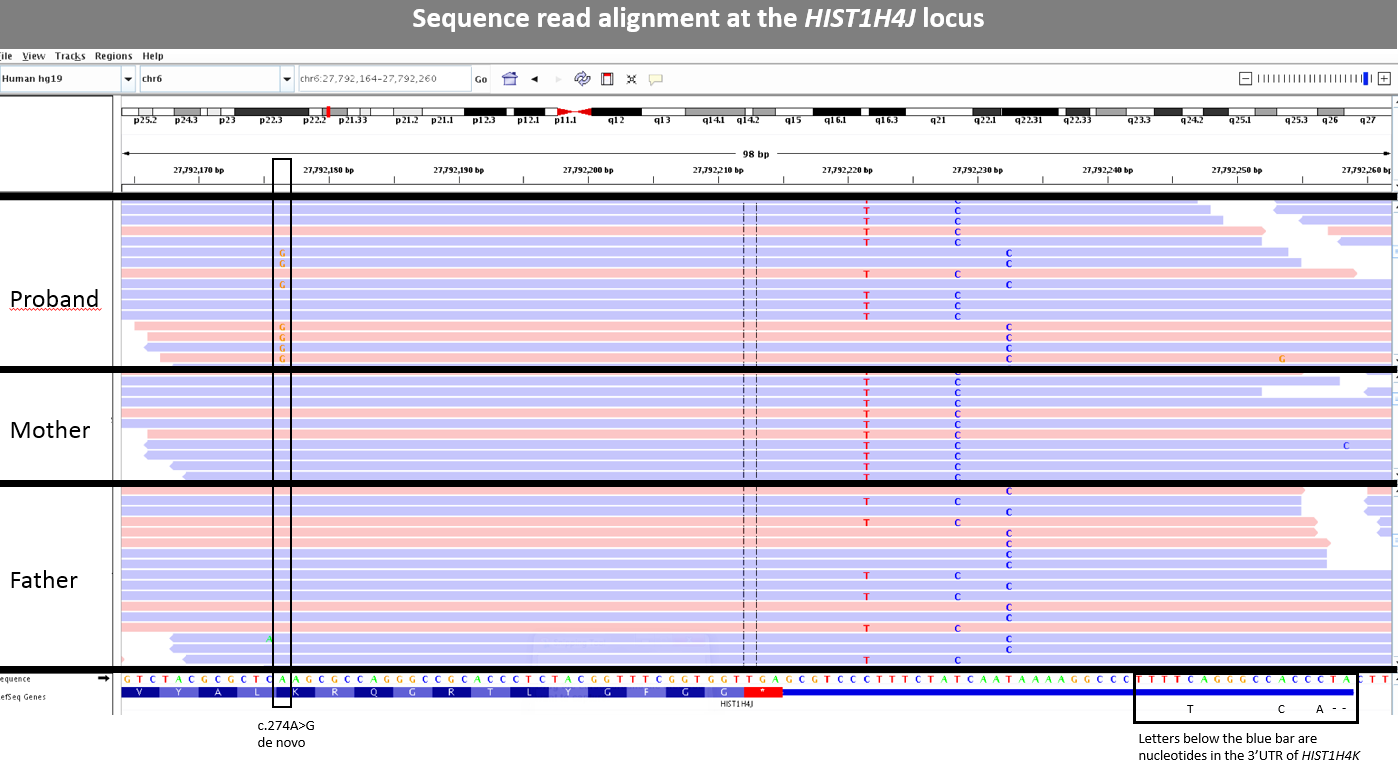


**c**


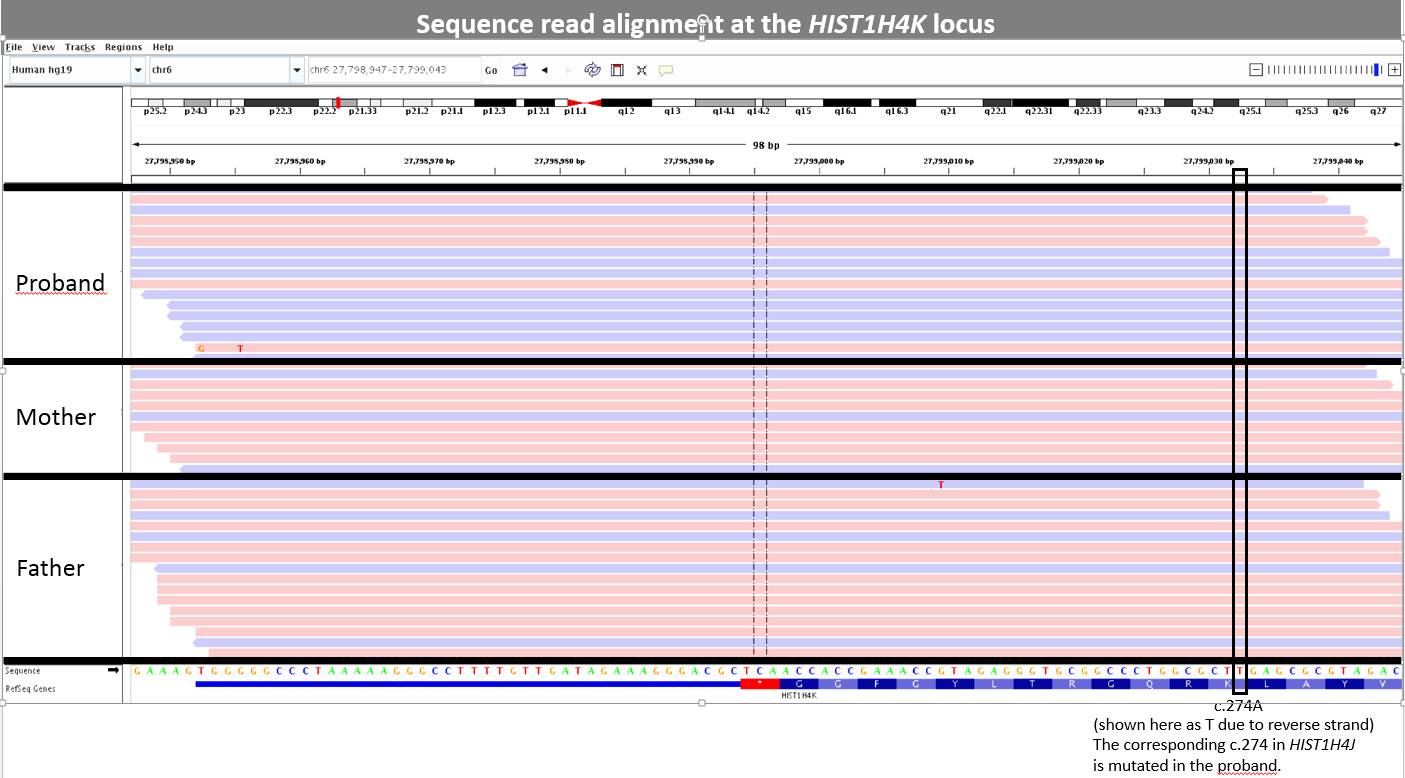


**Figure S2. Visual inspection of next-gen sequencing reads to validate the c.274A>G de novo variant (a)** Clustal Omega alignment of cDNA sequences of *HIST1H4J* and *HIST1H4K* showing identical open reading frames of both genes. Differences in sequences at the 3’UTR are highlighted in yellow. Bolded and enlarged letter “A” in red font is the location of c.274A>G p.(K91E) variant identified in the proband. **(b)** Sequence reads from trio WES in the bam file format aligned to the *HIST1H4J* locus. Pink bars represent sequence reads in forward direction whereas blue bars are reads in the opposite direction. The c.274A>G variant is present in the proband but not in his parents, suggesting the variant arose *de novo*. Moreover, reads containing c.274G allele have *HIST1H4J*-specifiec sequence in the 3-UTR confirming correct alignment of sequence reads. **(c)** Sequencing reads at the *HIST1H4K* locus have *HIST1H4K-*specific sequence in the 3’-UTR. Proband and his parents are homozygous for the reference allele at position c.274 of *HIST1H4K*.
